# Association between red blood cell distribution width-to-albumin ratio and the prognosis in patients with autoimmune encephalitis: a retrospective cohort study

**DOI:** 10.3389/fneur.2023.1276026

**Published:** 2024-01-11

**Authors:** Dan Li, Ali Yang, Mingrong Xia, Kai Ma, Jiewen Zhang, Yang Guo, Weizhou Zang

**Affiliations:** ^1^Department of Neurology, Henan Provincial People's Hospital, People's Hospital of Zhengzhou University, People's Hospital of Henan University, Zhengzhou, China; ^2^Department of Neurosurgery, Henan Provincial People's Hospital, People's Hospital of Zhengzhou University, People's Hospital of Henan University, Zhengzhou, China

**Keywords:** RAR, autoimmune encephalitis, modified Rankin Scale, red blood cell distribution width, albumin

## Abstract

**Aim:**

Red blood cell distribution width-to-albumin ratio (RAR) is a combined new indicator reflecting immunology and has been reported to predict the prognosis of inflammation-related diseases and brain diseases. However, the association and predictive value of RAR in the prognosis of patients with autoimmune encephalitis (AE) has not been reported.

**Methods:**

This was a retrospective cohort study, and data were collected from the Henan Provincial People’s Hospital. RAR was categorized according to quartile. The prognosis was assessed using the modified Rankin Scale (mRS), and an mRS score of ≥3 was defined as a poor prognosis. The logistical regression model was used to explore the association between RAR and the prognosis, with results reported as odds ratio (OR) and 95% confidence interval (CI). The predictive value of RAR was evaluated by calculating the area under the receiving operating curve (AUC), sensitivity, specificity, and accuracy.

**Results:**

A total of 175 eligible patients were included for analysis, and 51 patients were identified as having poor prognosis. After adjusting age, cancer, other diseases, histological subtype, antiepileptic therapy, anti-tumor treatment, ICU treatment, and length of stay, RAR in the highest quartile (Q4) was found to be significantly associated with the high odds of poor prognosis (OR = 5.63, 95%CI: 1.98–16.02) compared to RAR in the lowest quartile (Q1). In addition, RAR was identified as a predictor for the prognosis of AE patients (AUC = 0.660, 95%CI: 0.574–0.746).

**Conclusion:**

This study found the close association and predictive value of RAR for the prognosis of AE patients, indicating that RAR might help clinicians identify high-risk populations.

## Introduction

Autoimmune encephalitis (AE) is a severe inflammatory disorder of the brain, which is mediated by autoimmune mechanisms and characterized by prominent neuropsychiatric symptoms (1). AE is considered to be related to antibodies against neuronal cell surface proteins, ion channels, or receptors, and accounts for approximately 20% of all adult encephalitis cases (1, 2). As a heterogeneous disease, AE is characterized by complex clinical manifestations and frequent complications (3, 4). Although approximately 80% of AE patients recover well after immunotherapy, there are still some patients not responding to the treatments (3, 4). Therefore, exploring biomarkers related to the prognosis of AE patients is of great clinical significance.

Immunity and inflammation are important mechanisms for the onset and progression of AE (5, 6). Red blood cell distribution width (RDW) is a biomarker reflecting the variation in red blood cell size, and a high value of RDW is related to inflammation (7). Increasing RDW value has been reported to be associated with the development and prognosis of many inflammation-related diseases including autoimmune diseases, such as acute kidney injury, autoimmune liver diseases, and autoimmune gastritis (8–10). However, the prognostic role of RDW has not been reported in AE.

Albumin (ALB) is also a biomarker reflecting inflammatory response and nutritional states, and the level of ALB is decreased in the inflammatory state (11). A study has shown that low concentration of serum ALB is significantly associated with the short-term and long-term adverse prognosis in AE patients (12). Existing studies believed that the ratio of RDW to ALB (RAR) was a combined new indicator that can reflect immunology and nutrition and predict the prognosis of inflammation-related diseases and brain diseases (13, 14). Previous studies have reported the association between regular inflammation indicators, such as neutrophil-to-lymphocyte ratio (NLR) and the prognosis of AE (1, 15). However, the association between RAR and the prognosis of AE patients has not been reported.

In this study, we aimed to explore the association between RDW or RAR and the prognosis of AE patients. For the purpose of identifying the optimal biomarker, we attempted to compare the predictive value of RDW and RAR with other regular inflammation indicators [NLR and platelet to lymphocyte (PLR) ratio] in the prognosis of AE patients.

## Methods

### Study design

Patients with AE diagnosed in the Henan Provincial People’s Hospital from January 2017 to February 2022 were enrolled in this study. This retrospective cohort study has been reviewed and approved by the Ethics Committee of the Henan Provincial People’s Hospital (Number: 2022-1-233). The need for written informed consent was waived by the Ethics Committee of the Henan Provincial People’s Hospital due to the retrospective nature of the study.

### Study population

Patients who met the following criteria were included: (1) those aged ≥16 years; (2) those with a positive serum and/or cerebrospinal fluid (CSF) antibody test for neuronal cell surface antibodies; and (3) those with completely preserved clinical data. Patients who met one of the following criteria were excluded: (1) those receiving immunotherapy before laboratory examination; (2) those with other autoimmune diseases; (3) those with respiratory tract infection, urinary system infection, or other infectious diseases at the time of admission; (4) those with a modified Rankin Scale (mRS) score of ≥2 points before the onset of AE; (5) those with infectious encephalitis, epidemic encephalitis, unexplained encephalitis, central nervous system tumors, demyelinating diseases, and other neurological diseases; (6) those not detecting RDW; (7) those not detecting albumin; and (8) those lost to follow-up. The follow-up period was 1 year after admission, and the follow-up was ended if patients died.

### Data collection and definition

Data were collected based on demographic characteristics, living habits, history of diseases, laboratory examination, cerebrospinal fluid examination, imaging examination, disease characteristics, treatment-related information, factors for poor prognosis in the prediction model reported by another study (reported model), and Clinical Assessment Scale in Autoimmune Encephalitis (CASE) score.

Demographic characteristics include age, sex, and body mass index (BMI). BMI was calculated as weight (kg)/height (m)^2^ and divided into underweight (< 18.5 kg/m^2^), normal weight (18.5 kg/m^2^ ≤ BMI < 24 kg/m^2^), overweight (24 kg/m^2^ ≤ BMI < 28 kg/m^2^), and obesity (≥ 28 kg/m^2^) (16).

Living habits contained drinking (no/yes) and smoking (no/yes).

History of diseases contained cancer and other diseases. Other diseases included diabetes, hypertension, cardiovascular diseases, hyperlipidemia, hyperthyroidism, viral myocarditis, peripheral facial paralysis, lower limb vein thrombosis, asthma, kidney stone disease, brain injury, hepatitis B, duodenal ulcer, myocardial ischemia, and erosive gastritis.

Laboratory examination included the routine blood test, liver function test, and renal function test. Routine blood indicators contained hemoglobin, red blood cells (RBC), white blood cells (WBC), platelets, neutrophils, lymphocytes, monocytes, and mean platelet volume (MPV). Liver function indicators contained alanine transaminase (ALT), aspartate aminotransferase (AST), gamma-glutamyltransferase (GGT), alkaline phosphatase (ALP), albumin, and globulin. Renal function indicators contained blood urea nitrogen (BUN), creatinine, and uric acid.

Cerebrospinal fluid examination contained increased cerebrospinal fluid pressure (no/yes), increased cerebrospinal fluid protein (no/yes), and increased cerebrospinal fluid WBC (no/yes).

Imaging examination contained the results of magnetic resonance imaging (MRI) and divided into normal and abnormal.

Disease characteristics contained Glasgow coma score (GCS), clinical features (mental and behavioral abnormalities, limb numbness and weakness, autonomic dysfunction, epileptic seizure, and others), and histological subtype [N-methyl-D-aspartic acid receptor antibody (anti-NMDAR), paraneoplastic, and others]. The GCS was used to assess the degree of coma and calculated based on the sum of the scores for the functions of eye opening, verbal response, and motor response, with a lower score indicating a higher degree of coma (17).

Treatment-related information contained immunotherapy regimen (first-line agent, first-line combined second-line agents, second-line agent, and none), antiepileptic therapy (no/yes), antipsychotic therapy (no/yes), anti-tumor treatment (no/yes), intensive care unit (ICU) treatment (no/yes), and length of stay.

Factors in the reported model included viral prodrome (no/yes), memory dysfunction (no/yes), consciousness impairment (no/yes), and autonomic dysfunction (no/yes) (4).

The CASE score included nine items: seizure, memory dysfunction, psychiatric symptoms, consciousness, language problems, dyskinesia/dystonia, gait instability and ataxia, brainstem dysfunction, and muscle weakness (18). In this study, one point was assigned for the occurrence of one item.

### Exposures

Exposures were RDW, RAR, PLR, and NLR, which were divided according to quartile. RDW was divided into Q1 (RDW < 41 fL), Q2 (41 fL ≤ RDW < 43 fL), Q3 (43 fL ≤ RDW < 45 fL), and Q4 (RDW ≥ 45 fL). RAR was divided into Q1 (< 1.00), Q2 (1.00–1.09), Q3 (1.09–1.22), and Q4 (≥ 1.22). PLR was divided into Q1 (< 108.40), Q2 (108.40–134.33), Q3 (134.33–177.52), and Q4 (≥ 177.52). NLR was divided into Q1 (< 1.85), Q2 (1.85–2.69), Q3 (2.69–4.20), and Q4 (≥ 4.20).

### Outcome

The outcome was 1-year mRS. The mRS score contained six categories: no disability (mRS = 0); with no significant functional impairment and could complete all daily duties and activities despite some symptoms (mRS 1 point); with a mild disability that is unable to complete all previous activities but could look after one’s own affairs without assistance (mRS 2 points); with a moderate disability that required some assistance in daily life but could walk independently (mRS 3 points); with a moderate–severe disability that required others to take care of them and could not walk independently (mRS 4 points); a severe disability that required intensive care (mRS 5 points); and death (mRS 6 points) (19). Patients with an mRS score of 0–2 were defined as good prognosis, and patients with an mRS score of 3–6 were defined as poor prognosis (19).

### Statistical analysis

The continuous data in normal distribution were expressed as mean ± standard deviation (Mean ± SD), and the t-test was used to compare the differences between the two groups. The continuous data in skew distribution were expressed as median and quartile [M (Q1, Q3)], and the Wilcoxon rank sum test was used to compare the differences between the two groups. The categorized data were expressed as number and percentage [*n* (%)], and the chi-squared test or Fisher’s exact test was used to compare the difference between the two groups. The missing data were processed using random forest imputation.

Univariate and multivariate logistical regression models were used to explore the association between RDW, RAR, PLR, NLR, and the prognosis (mRS), and results were shown as odds ratio (OR) with 95% confidence interval (CI). The potential covariables were selected using the univariate logistical regression model. Restricted cubic spline (RCS) was used to assess the association between RAR and the odds of poor prognosis. The predictive value of RAR in the prognosis of AE patients was assessed by calculating the area under the receiving operating curve (AUC), sensitivity, specificity, and accuracy. A visual calibration plot was used to evaluate calibration.

To further assess the predictive value of RAR, we assessed the predictive performance of model based on CASE, CASE + RAR, reported model, and reported model + RAR. The net reclassification improvement (NRI) and integrated discrimination improvement (IDI) were computed to assess the performance improvement of the CASE + RAR-based or reported model + RAR-based models over the CASE-based or reported model-based models (20). Positive values of IDI indicated better performance in the new model than in the reference model. The statistical analysis was performed using Python 3.9.12 (Python Software Foundation, Delaware, United States) and SAS 9.4 (SAS Institute Inc., Cary, NC, United States). A *p*-value of <0.05 was considered to be statistically significant.

## Results

### Selection and characteristics of the study population

**Figure 1:**
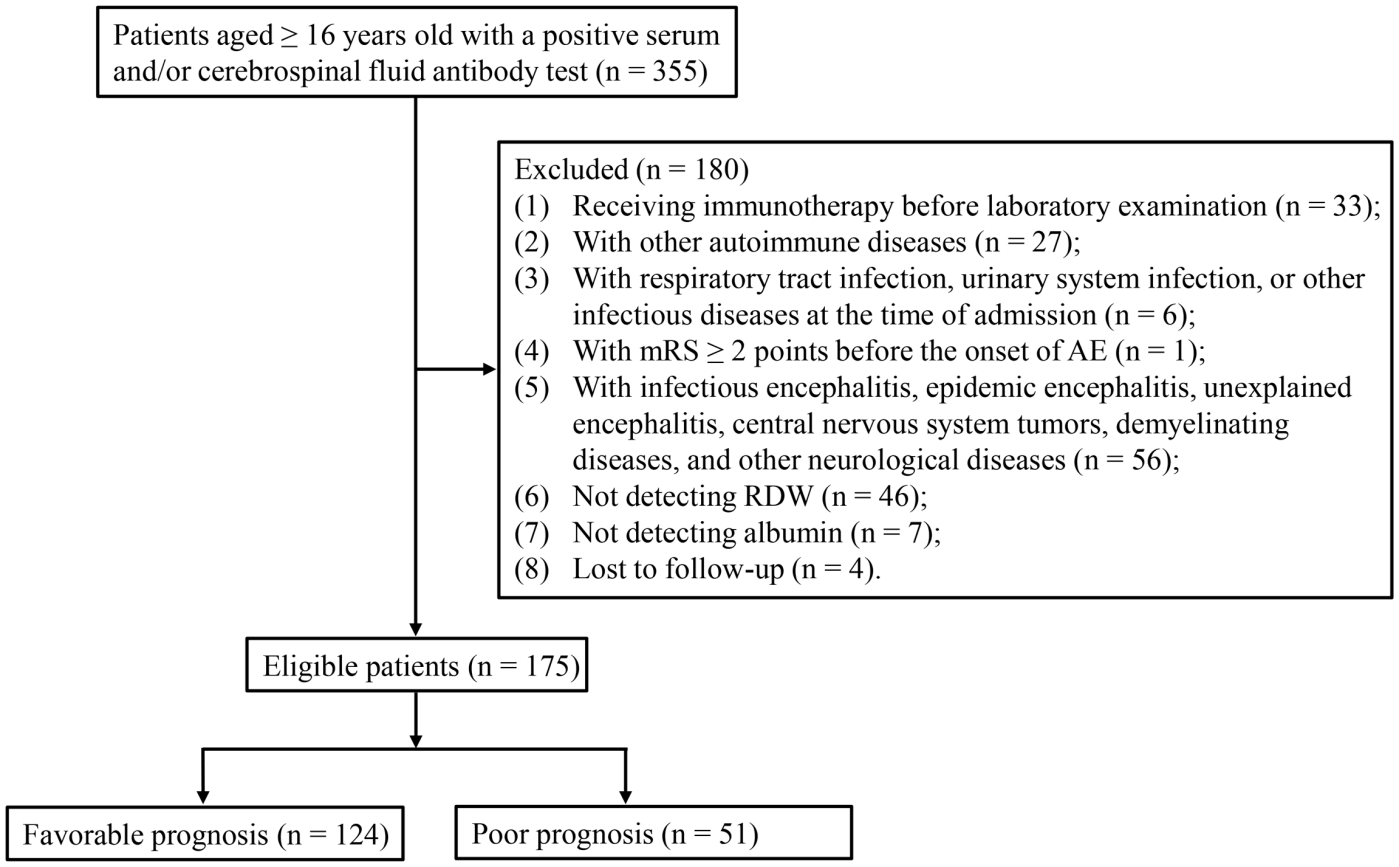
Flowchart for the selection of participants.

A total of 355 patients aged ≥16 years old with a positive serum and/or cerebrospinal fluid antibody test were included in this study. Of these, 180 patients were excluded due to receiving immunotherapy before laboratory examination (*n* = 33), with other autoimmune diseases (*n* = 27), with respiratory tract infection, urinary system infection, or other infectious diseases at the time of admission (*n* = 6), with mRS ≥ 2 points before the onset of AE (*n* = 1), with infectious encephalitis, epidemic encephalitis, unexplained encephalitis, central nervous system tumors, demyelinating diseases, and other neurological diseases (*n* = 56), not detecting RDW (*n* = 46), not detecting albumin (*n* = 7), and lost to follow-up (*n* = 4). Finally, 175 eligible patients were included, with 124 patients in the favorable prognosis group and 51 patients in the poor prognosis (Figure 1). Sensitivity analysis showed that there was no statistical significance between before imputation and after imputation (Supplementary Table S1).

The baseline information of 175 patients is shown in Table 1. This study consisted of 96 male (54.86%) and 79 female (45.14%) subjects with a median age of 54 (31, 65) years. There was statistical significance in age, cancer, other diseases, ALT, clinical features, histological subtype, antiepileptic therapy, anti-tumor treatment, ICU treatment, follow-up time, and RAR between the favorable prognosis group and the poor prognosis group.

**Table 1:** Characteristics of the favorable prognosis group and poor prognosis group.

Variables	Total (*n* = 175)	1-year mRS		Statistics	*p*
		Favorable prognosis (*n* = 124)	Poor prognosis (*n* = 51)		
Age, years, M (Q_1_, Q_3_)	54.00 (31.00, 65.00)	47.00 (26.00, 60.50)	63.00 (51.00, 71.00)	*Z* = 4.689	<0.001
Sex, *n* (%)				χ^2^ = 0.000	0.994
Male	96 (54.86)	68 (54.84)	28 (54.90)		
Female	79 (45.14)	56 (45.16)	23 (45.10)		
BMI, *n* (%)				–	0.218
Normal	98 (56.00)	64 (51.61)	34 (66.67)		
Underweight	8 (4.57)	5 (4.03)	3 (5.88)		
Overweight	52 (29.71)	41 (33.06)	11 (21.57)		
Obesity	17 (9.71)	14 (11.29)	3 (5.88)		
Drinking, *n* (%)				χ^2^ = 0.000	0.984
No	134 (76.57)	95 (76.61)	39 (76.47)		
Yes	41 (23.43)	29 (23.39)	12 (23.53)		
Smoking, *n* (%)				χ^2^ = 1.594	0.207
No	125 (71.43)	92 (74.19)	33 (64.71)		
Yes	50 (28.57)	32 (25.81)	18 (35.29)		
Cancer, *n* (%)				χ^2^ = 32.391	< 0.001
No	142 (81.14)	114 (91.94)	28 (54.90)		
Yes	33 (18.86)	10 (8.06)	23 (45.10)		
Other diseases, *n* (%)				χ^2^ = 6.270	0.012
No	91 (52.00)	72 (58.06)	19 (37.25)		
Yes	84 (48.00)	52 (41.94)	32 (62.75)		
Hemoglobin, g/L, Mean ± SD	131.19 ± 15.58	132.42 ± 15.47	128.22 ± 15.60	*t* = 1.63	0.105
RBC, 10^12/L, Mean ± SD	4.27 ± 0.51	4.30 ± 0.52	4.20 ± 0.47	*t* = 1.22	0.226
WBC, 10^9/L, M (Q_1_, Q_3_)	7.01 (5.72, 8.64)	6.88 (5.66, 8.60)	7.26 (5.95, 9.05)	Z = 1.052	0.293
Platelet, 10^9/L, Mean ± SD	234.18 ± 67.69	235.70 ± 68.50	230.49 ± 66.19	*t* = 0.46	0.645
Neutrophil, 10^9/L, M (Q_1_, Q_3_)	4.53 (3.25, 6.33)	4.34 (3.14, 6.30)	4.99 (3.40, 6.75)	Z = 1.307	0.191
Lymphocyte, 10^9/L, M (Q_1_, Q_3_)	1.64 (1.25, 2.12)	1.70 (1.26, 2.26)	1.58 (1.16, 1.95)	Z = −1.149	0.250
Monocyte, 10^9/L, M (Q_1_, Q_3_)	0.40 (0.32, 0.61)	0.41 (0.33, 0.58)	0.40 (0.32, 0.68)	Z = 0.841	0.400
MPV, fL, Mean ± SD	10.31 ± 1.06	10.37 ± 1.12	10.17 ± 0.92	*t* = 1.12	0.266
ALT, U/L, M (Q_1_, Q_3_)	19.00 (12.40, 27.50)	21.55 (13.65, 28.60)	15.00 (11.10, 20.90)	Z = −2.558	0.011
AST, U/L, M (Q_1_, Q_3_)	18.90 (15.00, 24.00)	18.95 (15.05, 25.10)	18.70 (15.00, 22.40)	Z = −0.186	0.853
GGT, μmol/L, M (Q_1_, Q_3_)	23.10 (15.90, 35.90)	23.40 (16.00, 35.95)	21.30 (15.00, 35.00)	Z = −0.688	0.491
ALP, U/L, Mean ± SD	67.44 ± 21.27	66.40 ± 22.04	69.99 ± 19.24	*t* = −1.01	0.312
Albumin, g/L, Mean ± SD	39.14 ± 3.92	39.50 ± 3.95	38.27 ± 3.73	*t* = 1.90	0.059
Globulin, g/L, Mean ± SD	25.28 ± 4.06	24.96 ± 3.83	26.05 ± 4.51	*t* = −1.62	0.107
BUN, mmol/L, Mean ± SD	4.73 ± 1.49	4.71 ± 1.47	4.79 ± 1.54	*t* = −0.32	0.747
Creatinine, μmol/L, Mean ± SD	55.72 ± 13.41	56.11 ± 13.01	54.76 ± 14.42	*t* = 0.60	0.547
Uric acid, μmol/L, M (Q_1_, Q_3_)	246.00 (192.00, 305.00)	252.00 (198.00, 308.00)	230.00 (184.00, 288.00)	*Z* = −1.914	0.056
Increased CSF pressure, *n* (%)				*χ*^2^ = 2.405	0.121
No	134 (76.57)	91 (73.39)	43 (84.31)		
Yes	41 (23.43)	33 (26.61)	8 (15.69)		
Increased CSF protein, *n* (%)				*χ*^2^ = 1.423	0.233
No	98 (56.00)	73 (58.87)	25 (49.02)		
Yes	77 (44.00)	51 (41.13)	26 (50.98)		
Increased CSF WBC, *n* (%)				*χ*^2^ = 0.000	0.983
No	86 (49.14)	61 (49.19)	25 (49.02)		
Yes	89 (50.86)	63 (50.81)	26 (50.98)		
Craniocerebral MRI results, *n* (%)				*χ*^2^ = 0.125	0.724
Normal	89 (50.86)	62 (50.00)	27 (52.94)		
Abnormal	86 (49.14)	62 (50.00)	24 (47.06)		
GCS score, Mean ± SD	14.42 ± 1.87	14.60 ± 1.34	14.00 ± 2.75	*t* = 1.48	0.144
Clinical features, *n* (%)				–	0.039
Mental and behavioral abnormalities	32 (18.29)	23 (18.55)	9 (17.65)		
Limb numbness and weakness	21 (12.00)	11 (8.87)	10 (19.61)		
Autonomic dysfunction	15 (8.57)	9 (7.26)	6 (11.76)		
Epileptic seizure	84 (48.00)	68 (54.84)	16 (31.37)		
Others	18 (10.29)	10 (8.06)	8 (15.69)		
Histological subtype, *n* (%)				χ^2^ = 7.377	0.025
Anti-NMDAR	48 (27.43)	41 (33.06)	7 (13.73)		
Paraneoplastic	55 (31.43)	34 (27.42)	21 (41.18)		
Others	72 (41.14)	49 (39.52)	23 (45.10)		
Immunotherapy regimen, n (%)				–	0.147
First-line agent	89 (50.86)	59 (47.58)	30 (58.82)		
First-line + second-line agents	54 (30.86)	44 (35.48)	10 (19.61)		
Second-line agent	1 (0.57)	1 (0.81)	0 (0.00)		
None	31 (17.71)	20 (16.13)	11 (21.57)		
Antiepileptic therapy, *n* (%)				*χ*^2^ = 8.048	0.005
No	84 (48.00)	51 (41.13)	33 (64.71)		
Yes	91 (52.00)	73 (58.87)	18 (35.29)		
Antipsychotic therapy, *n* (%)				*χ*^2^ = 0.132	0.716
No	110 (62.86)	79 (63.71)	31 (60.78)		
Yes	65 (37.14)	45 (36.29)	20 (39.22)		
Anti-tumor treatment, *n* (%)				*χ*^2^ = 16.272	< 0.001
No	154 (88.00)	117 (94.35)	37 (72.55)		
Yes	21 (12.00)	7 (5.65)	14 (27.45)		
ICU treatment, *n* (%)				*χ*^2^ = 6.241	0.012
No	154 (88.00)	114 (91.94)	40 (78.43)		
Yes	21 (12.00)	10 (8.06)	11 (21.57)		
Length of stay, days, M (Q_1_, Q_3_)	13.00 (10.00, 18.00)	13.00 (9.00, 17.00)	14.00 (10.00, 21.00)	*Z* = 1.849	0.064
Viral prodrome, *n* (%)				*χ*^2^ = 0.913	0.339
No	119 (68.00)	87 (70.16)	32 (62.75)		
Yes	56 (32.00)	37 (29.84)	19 (37.25)		
Memory dysfunction, *n* (%)				–	0.581
No	171 (97.71)	122 (98.39)	49 (96.08)		
Yes	4 (2.29)	2 (1.61)	2 (3.92)		
Consciousness impairment, *n* (%)				*χ^2^* = 0.470	0.493
No	133 (76.00)	96 (77.42)	37 (72.55)		
Yes	42 (24.00)	28 (22.58)	14 (27.45)		
Autonomic dysfunction, *n* (%)				*χ*^2^ = 0.385	0.535
No	139 (79.43)	100 (80.65)	39 (76.47)		
Yes	36 (20.57)	24 (19.35)	12 (23.53)		
CASE, score, M (Q_1_, Q_3_)	1.00 (1.00, 2.00)	1.00 (1.00, 2.00)	2.00 (1.00, 2.00)	*Z* = 0.883	0.377
Follow time, days, M (Q_1_, Q_3_)	365.00 (365.00, 365.00)	365.00 (365.00, 365.00)	365.00 (154.00, 365.00)	Z = −7.773	< 0.001
RDW, *n* (%)				*χ*^2^ = 3.862	0.277
Q1	34 (19.43)	27 (21.77)	7 (13.73)		
Q2	45 (25.71)	34 (27.42)	11 (21.57)		
Q3	41 (23.43)	29 (23.39)	12 (23.53)		
Q4	55 (31.43)	34 (27.42)	21 (41.18)		
RAR, *n* (%)				*χ*^2^ = 12.533	0.006
Q1	43 (24.57)	37 (29.84)	6 (11.76)		
Q2	44 (25.14)	33 (26.61)	11 (21.57)		
Q3	44 (25.14)	31 (25.00)	13 (25.49)		
Q4	44 (25.14)	23 (18.55)	21 (41.18)		
PLR, *n* (%)				*χ*^2^ = 4.724	0.193
Q1	44 (25.14)	33 (26.61)	11 (21.57)		
Q2	43 (24.57)	34 (27.42)	9 (17.65)		
Q3	44 (25.14)	26 (20.97)	18 (35.29)		
Q4	44 (25.14)	31 (25.00)	13 (25.49)		
NLR, *n* (%)				*χ*^2^ = 4.224	0.238
Q1	44 (25.14)	36 (29.03)	8 (15.69)		
Q2	43 (24.57)	31 (25.00)	12 (23.53)		
Q3	44 (25.14)	29 (23.39)	15 (29.41)		
Q4	44 (25.14)	28 (22.58)	16 (31.37)		

BMI, body mass index; RBC, red blood cell; WBC, white blood cell; MPV, mean platelet volume; ALT, alanine transaminase; AST, aspartate aminotransferase; GGT, gamma-glutamyltransferase; ALP, alkaline phosphatase; BUN, blood urea nitrogen; CSF, cerebrospinal fluid; MRI, magnetic resonance imaging; GCS, Glasgow coma score; NMDAR, N-methyl-D-aspartic acid receptor; ICU, intensive care unit; CASE, Clinical Assessment Scale in Autoimmune Encephalitis; RDW, red blood cell distribution width; RAR, red blood cell distribution width to albumin ratio; PLR, platelet-to-lymphocyte ratio; NLR, neutrophil-to-lymphocyte ratio.

Other diseases included diabetes, hypertension, cardiovascular diseases, hyperlipidemia, hyperthyroidism, viral myocarditis, peripheral facial paralysis, lower limb vein thrombosis, asthma, kidney stone disease, brain injury, hepatitis B, duodenal ulcer, myocardial ischemia, and erosive gastritis.

### Association between RDW, RAR, PLR, NLR, and the prognosis of AE patients

Supplementary Table S2 shows that age (OR = 1.05, 95% CI: 1.03–1.07), cancer (OR = 9.36, 95%CI: 4.00–21.90), other diseases (OR = 2.33, 95% CI: 1.19–4.56), histological subtype (paraneoplastic: OR = 3.62, 95% CI: 1.37–9.53; others: OR = 2.75, 95% CI: 1.07–7.05), antiepileptic therapy (OR = 0.38, 95% CI: 0.19–0.75), anti-tumor treatment (OR = 6.32, 95% CI: 2.37–16.85), ICU treatment (OR = 3.13, 95% CI: 1.24–7.94), and length of stay (OR = 1.04, 95% CI: 1.01–1.07) were identified as the covariables.

**Figure 2:**
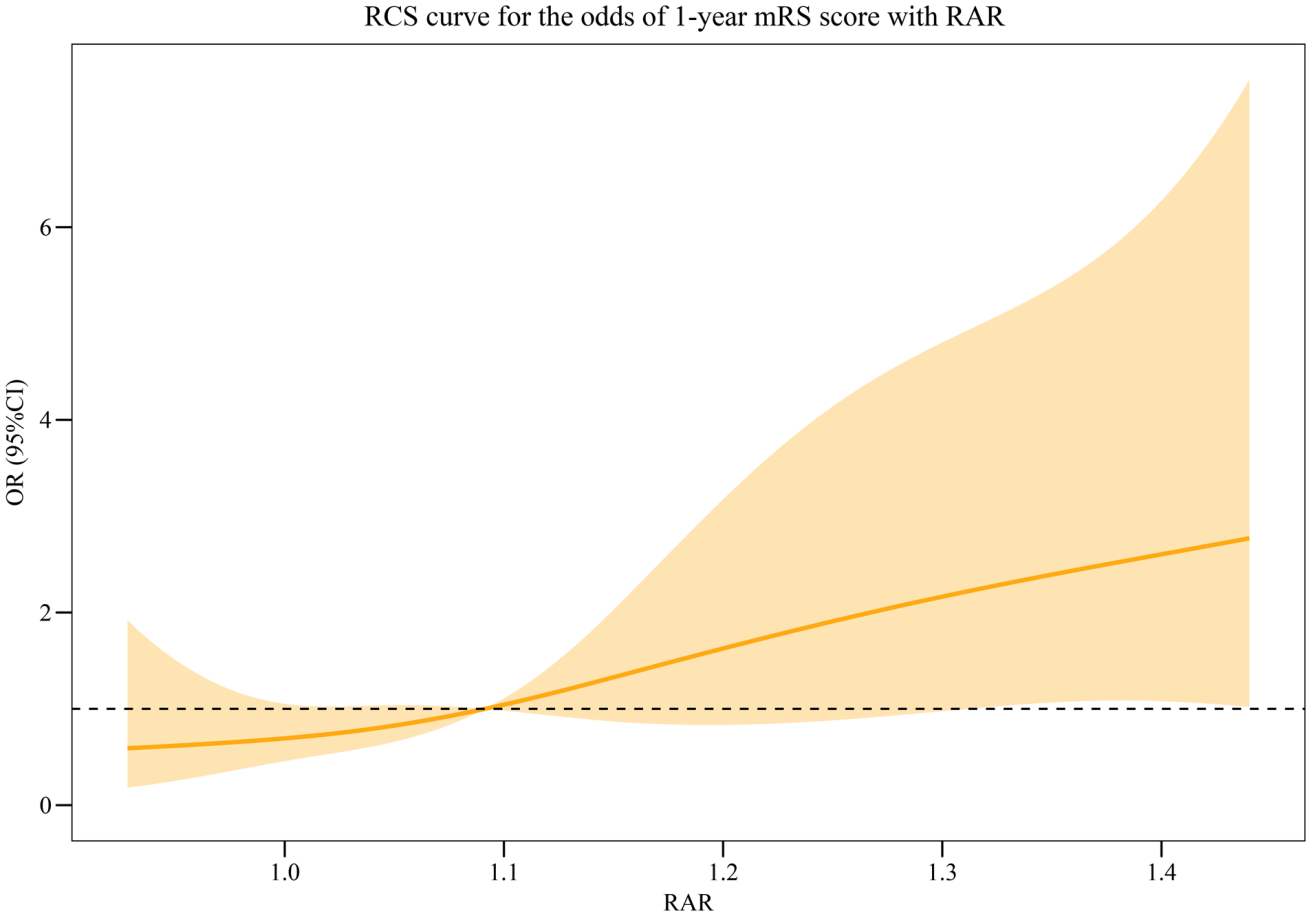
RCS curve for the association between RAR and the odds of poor prognosis in AE patients.

Table 2 displays that the highest quartile (Q4) of RAR was significantly associated with the higher odds of poor prognosis (OR = 5.63, 95% CI: 1.98–16.02) compared to the lowest quartile (Q1) of RAR. After adjusting age, cancer, other disease, histological subtype, antiepileptic therapy, anti-tumor treatment, ICU treatment, and length of stay, we also found a positive association between RAR in the Q4 and the poor prognosis (OR = 3.38, 95% CI: 1.01–11.37). The RCS curve showed that the odds of poor prognosis in AE patients increased with the increase of RAR (Figure 2). There was no difference between RDW, PLR, NLR, and the prognosis of AE patients (all *p* > 0.05).

**Table 2:** Association between RDW, RAR, PLR, NLR, and 1-year mRS score.

Variables	Univariable model		Multivariable model	
	OR (95% CI)	*p*	OR (95% CI)	*p*
RDW				
Q1	Ref		Ref	
Q2	1.25 (0.43–3.65)	0.686	1.47 (0.41–5.28)	0.550
Q3	1.60 (0.55–4.65)	0.392	2.23 (0.61–8.18)	0.226
Q4	2.38 (0.88–6.43)	0.087	1.62 (0.47–5.57)	0.443
RAR				
Q1	Ref		Ref	
Q2	2.06 (0.68–6.17)	0.199	2.01 (0.55–7.39)	0.292
Q3	2.59 (0.88–7.60)	0.084	1.21 (0.34–4.25)	0.768
Q4	5.63 (1.98–16.02)	0.001	3.38 (1.01–11.37)	0.049
PLR				
Q1	Ref		Ref	
Q2	0.79 (0.29–2.16)	0.652	1.06 (0.32–3.50)	0.924
Q3	2.08 (0.84–5.16)	0.115	2.45 (0.80–7.50)	0.117
Q4	1.26 (0.49–3.22)	0.632	1.40 (0.45–4.32)	0.563
NLR				
Q1	Ref		Ref	
Q2	1.74 (0.63–4.81)	0.284	1.32 (0.38–4.57)	0.666
Q3	2.33 (0.87–6.25)	0.094	1.69 (0.49–5.86)	0.409
Q4	2.57 (0.96–6.86)	0.059	2.20 (0.67–7.21)	0.194

Ref, reference; OR, odd ratio; CI, confidence interval; RDW, red blood cell distribution width; RAR, red blood cell distribution width to albumin ratio; PLR, platelet-to-lymphocyte ratio; NLR, neutrophil-to-lymphocyte ratio.

Univariable model: unadjusted model; Multivariable model: adjusted age, cancer, other disease, histological subtype, antiepileptic therapy, anti-tumor treatment, ICU treatment, and length of stay.

### Predictive value of RAR in the prognosis of AE patients

**Figure 3:**
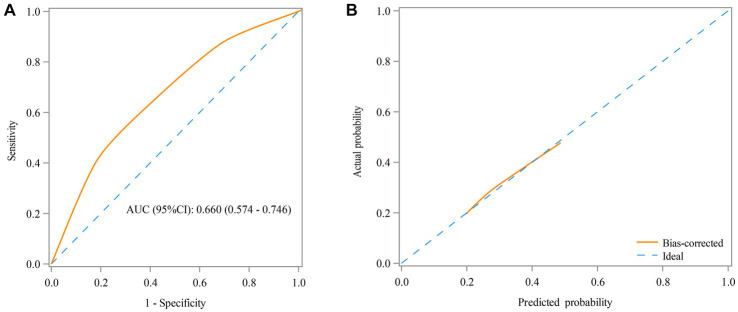
Receiver operating characteristic (ROC) curve **(A)** and calibration curve **(B)** for the predictive value of RAR in the prognosis of AE patients in the univariable model.

**Figure 4:**
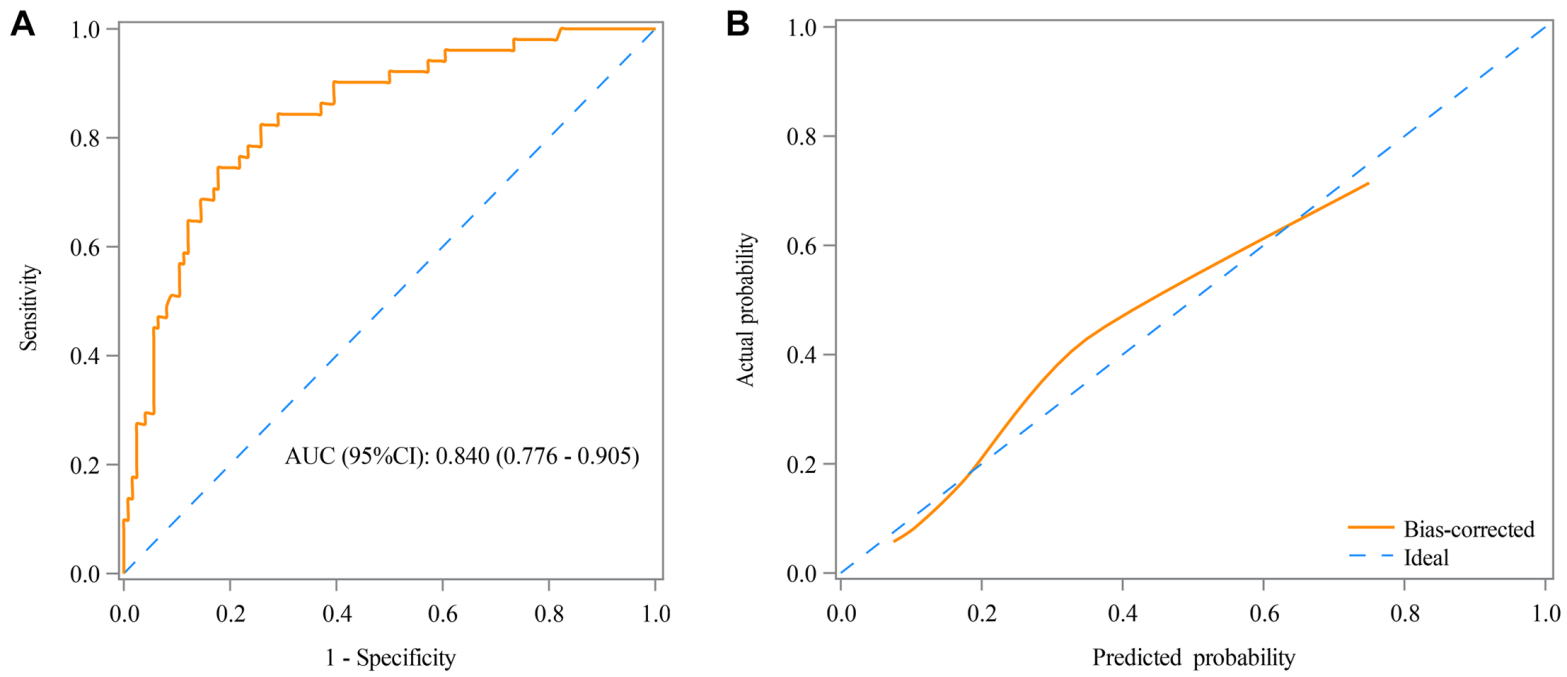
Receiver operating characteristic (ROC) curve **(A)** and calibration curve **(B)** for the predictive value of RAR in the prognosis of AE patients in the multivariable model.

Table 3 shows that the RAR was found as a predictor for the prognosis of AE patients, with an AUC of 0.660 (95% CI: 0.574–0.746) (Figure 3A) and an optimal cutoff value of 0.25 (sensitivity 66.7% and specificity 56.5%). Figure 3B demonstrates that the predicted probability of the poor prognosis fits well with the actual probability. For the multivariable model, the AUC was 0.840 (95% CI: 0.776–0.905) (Figure 4A), and the optimal cutoff value was 0.35 (sensitivity 74.5% and specificity 82.3%). The calibration plot demonstrated the good calibration of the multivariable model (Figure 4B).

**Table 3:** Predictive value of RAR in the prognosis of AE patients.

Model	Cutoff	AUC (95% CI)	Sensitivity (95% CI)	Specificity (95% CI)	Accuracy (95% CI)
Univariable	0.25	0.660 (0.574–0.746)	0.667 (0.537–0.796)	0.565 (0.477–0.652)	0.594 (0.522–0.667)
Multivariable	0.35	0.840 (0.776–0.905)	0.745 (0.625–0.865)	0.823 (0.755–0.890)	0.800 (0.741–0.859)

RAR, red blood cell distribution width to albumin ratio; AE, autoimmune encephalitis; AUC, the area under the receiving operating curve; CI, confidence interval.

Supplementary Table S3 lists the predictive performances of the risk prediction models based on CASE, CASE + RAR, reported model, reported model + RAR in terms of prognosis of AE patients. The CASE and CASE + RAR model for the poor prognosis of AE patients had an AUC of 0.540 (95% CI: 0.450–0.630) and 0.674 (95% CI: 0.586–0.761), respectively. When we compared the two models, the CASE + RAR model had a significantly better reclassification performance (NRI: 0.524, *p* = 0.001) and integrated discrimination (IDI = 0.076, *p* < 0.001) than the CASE model. The reported model and reported model + RAR model for the poor prognosis of AE patients had an AUC of 0.674 (95% CI: 0.592–0.757) and 0.725 (95% CI: 0.647–0.803), respectively. When we compared the two models, the reported model + RAR model had a significantly better reclassification performance (NRI: 0.453, *p* = 0.003) and integrated discrimination (IDI = 0.062, *p* = 0.002) than the reported model. These findings indicated that RAR significantly improved the predictive performance of CASE score and reported model.

## Discussion

Autoimmune encephalitis (AE) is an autoimmune inflammatory disease that targets the surface of neuronal cell or synaptic proteins in the central nervous system (1). RAR was a combined indicator reflecting immunology and nutrition and has been reported to predict the prognosis of several inflammation-related diseases and brain diseases (13, 14). Some regular inflammation indicators have been found to be associated with the prognosis of AE (1, 15). In this study, we retrospectively explored the association between RAR and the prognosis of AE patients. The results displayed that the high level of RAR was associated with the high odds of poor prognosis in AE patients. The AUC of RAR for the poor prognosis in AE patients was 0.660. The combination of the RAR with the CASE score and reported model significantly improved their prognostic predictive performance.

RDW increases in response to inflammatory stimuli (21). It has been reported that the increase of RDW is associated with poor prognosis of many inflammatory diseases, such as sepsis, pneumonia, and acute respiratory distress syndrome (22–24). In addition, RDW was found to be associated with autoimmune diseases (25–27). The potential mechanism was that inflammation activity inhibited iron metabolism and erythropoietin production, and more immature cells were released into the bloodstream, which led to an increase in the RDW level (27). Another potential explanation was that the inflammatory cytokines may inhibit the maturation of erythrocytes and accelerate the entry of immature and larger volume reticulocytes into the peripheral circulation, therefore leading to the increase of RDW (27). ALB was a kind of multifunctional non-glycosylated plasma protein, which could downregulate the expression and transport of inflammatory factors and decrease the inflammatory cascade reactions (28). The microvascular permeability and ALB escape were increased in an inflammatory state, which enlarged the interstitial space and increased the distribution volume of ALB (29). Moreover, the half-life of ALB was shortened in an inflammatory state, which reduced the total mass of ALB (29). The ALB level was found to be significantly lower in AE patients than in healthy controls (30). Jang et al. have reported that ALB may be a prognostic indicator in AE patients (12). Low ALB was associated with the higher severity of AE patients, and mRS in the low ALB group improved more slowly than in the high ALB group (12). RAR combined both RDW and ALB and reflected the state of the systemic immune response and inflammatory response (12). In this study, we found that high RAR was associated with higher odds of poor prognosis in AE patients.

Our study also explored the predictive performance of RAR in the prognosis of AE patients. RDW has been reported to independently predict the progression, disease activity, and prognosis of several autoimmune diseases, such as systemic lupus erythematosus, rheumatoid arthritis, and autoimmune hepatitis (25–27). ALB was also reported to independently predict the prognosis of patients with inflammation-induced diseases (31). The existing study has reported the predictive performance of RAR in the prognosis of patients with autoimmune diseases (32). Yin et al. have found that the predictive performance of RAR (AUC = 0.643) was very close to the acute physiology and chronic health evaluation (APACHE) II score (AUC = 0.699) and sequential organ failure assessment (SOFA) score (AUC = 0.691) for the prediction of the prognosis in patients with rheumatic diseases (32). In this study, we found that the AUC of RAR for the poor prognosis of AE patients was 0.660. In the multivariable model that RAR combined with some potential covariates (age, cancer, other diseases, histological subtype, antiepileptic therapy, anti-tumor treatment, ICU treatment, and length of stay), the AUC reached up to 0.840. The CASE score was designed to assess the severity of AE (18). A previous study also developed a model with good performance to predict the prognosis in AE (reported model) (4). Furthermore, when RAR was combined with the CASE score and reported model, the predictive performance of the CASE score and reported model was significantly improved. The findings revealed that the combination of the RAR with the two models enhanced their prognostic predictive power. Our study indicated that RAR might be a practical predictor to monitor the odds of poor prognosis in AE patients.

This study explores the association between RAR and the prognosis of AE patients and further explores the predictive value of RAR, which provides a reference for the selection of AE prognostic markers. However, there are several limitations. First, this is a retrospective cohort study performed in a single center, which has inevitable selection bias and recall bias. Multicenter studies are needed to further verify our findings. Second, we find the association between RAR and the prognosis of AE patients, while the causal relationship cannot be determined (33). In future, more studies are needed to explore the causality. Third, data after discharge, such as changes in treatment methods, are not collected, which may affect results. Fourth, RAR is calculated before the treatment. Whether dynamic RAR might better predict results, the poor prognosis of AE patients needs to be further explored. Fifth, the included patients are all from China, and the generality of our results in the other populations outside of China is unclear. Future studies performed in other countries are needed.

## Conclusion

In conclusion, our study found that high levels of RAR were significantly associated with high odds of poor prognosis in AE patients, and RAR was identified as a predictor for the prognosis of AE patients. Our findings indicated that RAR might be a practical biomarker to predict the odds of poor prognosis for AE patients, which may help clinicians identify high-risk populations.

## Data availability statement

The raw data supporting the conclusions of this article will be made available by the authors, without undue reservation.

## Ethics statement

The studies involving humans were approved by Henan Provincial People’s Hospital (Number: 2022-1-233). The studies were conducted in accordance with the local legislation and institutional requirements. The Ethics Committee/Institutional Review Board waived the requirement of written informed consent for participation from the participants or the participants' legal guardians/next of kin because retrospective nature of the study.

## Author contributions

DL: Conceptualization, Supervision, Writing – original draft, Writing – review & editing. AY: Data curation, Formal analysis, Funding acquisition, Investigation, Methodology, Writing – review & editing. MX: Data curation, Formal analysis, Investigation, Methodology, Writing – review & editing. KM: Data curation, Formal analysis, Investigation, Methodology, Writing – review & editing. JZ: Data curation, Formal analysis, Investigation, Methodology, Writing – review & editing. YG: Conceptualization, Writing – original draft, Writing – review & editing. WZ: Conceptualization, Writing – original draft, Writing – review & editing.
