# Supplementary material for: Association between red blood cell distribution width-to-albumin ratio and the prognosis in patients with autoimmune encephalitis: a retrospective cohort study
Source: Front Neurol. 2024 Jan 11;14:1276026. doi: 10.3389/fneur.2023.1276026 (PMC10808499; doi:10.3389/fneur.2023.1276026)
Supplement: Supplementary file 1 [file Data_Sheet_1.docx]

**Supplementary table S1 Sensitivity analysis for the imputation**

| **Variables** | **Before imputation (n = 175)** | **After imputation (n = 175)** | **Statistics** | ***P*** |
| --- | --- | --- | --- | --- |
| BUN, Mean ± SD | 4.73 ± 1.49 | 4.73 ± 1.49 | t = 0.02 | 0.988 |
| Creatinine, Mean ± SD | 55.67 ± 13.43 | 55.72 ± 13.41 | t = 0.03 | 0.974 |
| Uric acid, M (Q_1_, Q_3_) | 245.50 (192.00, 305.00) | 246.00 (192.00, 305.00) | Z = -0.037 | 0.971 |
| Increased CSF pressure, n (%) |  |  | χ^2^ = 0.189 | 0.664 |
| No | 120 (74.53) | 134 (76.57) |  |  |
| Yes | 41 (25.47) | 41 (23.43) |  |  |
| Increased CSF protein, n (%) |  |  | χ^2^ = 0.000 | 0.985 |
| No | 90 (55.90) | 98 (56.00) |  |  |
| Yes | 71 (44.10) | 77 (44.00) |  |  |
| Increased CSF WBC, n (%) |  |  | χ^2^ = 0.058 | 0.809 |
| No | 77 (47.83) | 86 (49.14) |  |  |
| Yes | 84 (52.17) | 89 (50.86) |  |  |
| Anti-tumor treatment, n (%) |  |  | χ^2^ = 0.000 | 0.984 |
| No | 153 (87.93) | 154 (88.00) |  |  |
| Yes | 21 (12.07) | 21 (12.00) |  |  |

BUN, blood urea nitrogen; CSF, cerebrospinal fluid; WBC, white blood cell.

**Supplementary table S2 Selection of covariables**

| **Variables** | **OR (95%CI)** | ***P*** |
| --- | --- | --- |
| Age | 1.05 (1.03-1.07) | < 0.001 |
| Sex |  |  |
| Male | Ref |  |
| Female | 1.00 (0.52-1.92) | 0.994 |
| BMI |  |  |
| Normal | Ref |  |
| Underweight | 1.13 (0.25-5.01) | 0.873 |
| Overweight | 0.51 (0.23-1.11) | 0.088 |
| Obesity | 0.40 (0.11-1.50) | 0.176 |
| Drinking |  |  |
| No | Ref |  |
| Yes | 1.01 (0.47-2.18) | 0.984 |
| Smoking |  |  |
| No | Ref |  |
| Yes | 1.57 (0.78-3.16) | 0.208 |
| Cancer |  |  |
| No | Ref |  |
| Yes | 9.36 (4.00-21.90) | < 0.001 |
| Other diseases |  |  |
| No | Ref |  |
| Yes | 2.33 (1.19-4.56) | 0.013 |
| Hemoglobin | 0.98 (0.96-1.00) | 0.107 |
| RBC | 0.67 (0.35-1.28) | 0.226 |
| WBC | 1.08 (0.94-1.24) | 0.266 |
| Monocyte | 3.44 (0.75-15.70) | 0.110 |
| MPV | 0.84 (0.61-1.15) | 0.265 |
| ALT | 0.98 (0.95-1.00) | 0.085 |
| AST | 1.00 (0.96-1.04) | 0.878 |
| GGT | 1.00 (0.98-1.02) | 0.654 |
| ALP | 1.01 (0.99-1.02) | 0.310 |
| Globulin | 1.07 (0.99-1.16) | 0.109 |
| BUN | 1.04 (0.83-1.29) | 0.746 |
| Creatinine | 0.99 (0.97-1.02) | 0.545 |
| Uric acid | 1.00 (0.99-1.00) | 0.067 |
| Increased CSF pressure |  |  |
| No | Ref |  |
| Yes | 0.51 (0.22-1.20) | 0.125 |
| Increased CSF protein |  |  |
| No | Ref |  |
| Yes | 1.49 (0.77-2.87) | 0.234 |
| Increased CSF WBC |  |  |
| No | Ref |  |
| Yes | 1.01 (0.52-1.93) | 0.983 |
| Craniocerebral MRI results |  |  |
| Normal | Ref |  |
| Abnormal | 0.89 (0.46-1.71) | 0.724 |
| GCS score | 0.86 (0.72-1.02) | 0.077 |
| Clinical feature |  |  |
| Mental and behavioral abnormalities | Ref |  |
| Consciousness disorder | 1.70 (0.24-11.95) | 0.592 |
| Limb numbness and weakness | 2.32 (0.73-7.35) | 0.152 |
| Autonomic dysfunction | 1.70 (0.47-6.18) | 0.418 |
| Epileptic seizure | 0.60 (0.23-1.54) | 0.291 |
| Others | 2.04 (0.61-6.84) | 0.246 |
| Histological subtype |  |  |
| Anti-NMDAR | Ref |  |
| Paraneoplastic | 3.62 (1.37-9.53) | 0.009 |
| Others | 2.75 (1.07-7.05) | 0.035 |
| Immunotherapy regimen |  |  |
| First-line agent | 0.92 (0.39-2.18) | 0.858 |
| First-line + second-line agents | 0.41 (0.15-1.13) | 0.085 |
| Second-line agent | - | 0.992 |
| None | Ref |  |
| Antiepileptic therapy |  |  |
| No | Ref |  |
| Yes | 0.38 (0.19-0.75) | 0.005 |
| Antipsychotic therapy |  |  |
| No | Ref |  |
| Yes | 1.13 (0.58-2.22) | 0.716 |
| Anti-tumor treatment |  |  |
| No | Ref |  |
| Yes | 6.32 (2.37-16.85) | < 0.001 |
| ICU treatment |  |  |
| No | Ref |  |
| Yes | 3.13 (1.24-7.94) | 0.016 |
| Length of stay | 1.04 (1.01-1.07) | 0.030 |

Ref, reference; OR, odds ratio; CI, confidence interval; BMI, body mass index; RBC, red blood cell; WBC, white blood cell; MPV, mean platelet volume; ALT, alanine transaminase; AST, aspartate aminotransferase; GGT, gamma-glutamyltransferase; ALP, alkaline phosphatase; BUN, blood urea nitrogen; CSF, cerebrospinal fluid; MRI, magnetic resonance imaging; GCS, Glasgow coma score; NMDAR, N-methyl-D-aspartic acid receptor; ICU, intensive care unit.

**Supplementary table S3 Comparison of the predictive performance between CASE and CASE+RAR, between Reported Model and Reported Model +RAR**

| **Model** | **AUC (95%CI)** | **NRI (*P* value)** | **IDI (*P* value)** |
| --- | --- | --- | --- |
| CASE | 0.540 (0.450-0.630) | Ref | Ref |
| CASE+RAR | 0.674 (0.586-0.761) | 0.524 (*P* = 0.001) | 0.076 (*P* < 0.001) |
| Reported Model | 0.674 (0.592-0.757) | Ref | Ref |
| Reported Model +RAR | 0.725 (0.647-0.803) | 0.453 (*P* = 0.003) | 0.062 (*P* = 0.002) |

CASE, Clinical Assessment Scale in Autoimmune Encephalitis; RAR, red blood cell distribution width to albumin ratio; AUC, the area under the receiving operating curve; NRI, net reclassification improvement; IDI, integrated discrimination improvement; CI, confidence interval.
